# Dr. Blegborough, on the Gout

**Published:** 1804-07-01

**Authors:** Ralph Blegborough

**Affiliations:** Margaret Street, Cavendish Square


### *To the Editors of the Medical and Physical Journal.*

GENTLEMEN,

I Was much pleased with the communications respecting Gout in your Journal, but am very sorry for the unlucky turn they have lately taken. On this account many may doubt the propriety of further agitating the question. I am of opinion, however, that the subject is too important to be so dismissed, and that it presents a wide field for the exertion of ingenuity; nor do I see why it may not be discussed without animosity.

Your sensible and facetious correspondent, *a Constant Reader*, has, I think, properly counterbalanced the hyperbolic praises of the indiscriminate use of cold water to parts labouring under gouty inflammation; and it is to be regretted, that, as he seems capable, he has not attempted to draw the line, and fix the limits, to which the abstraction of heat from such parts may with safety and propriety be carried.

" *That the gout is exclusively an inflammatory affection of the ligamentous and tendinous structure, that it is merely local and unknown as a constitutional complaint, &c.*" is an opinion opinion that I think with you, Gentlemen, "*will meet with general opposition.*"

It does not, I think, admit of doubt that the inflammation of gout is as specific, that is, as much *sui generis* as that of scrophula, syphilis, or cancer; nor do I think it requires the power of divination to predict the fate of a theory supported on the above, I will venture to say mistaken, opinion. The spurious *left-handed* inflammation of erysipelas is very analogous to that which takes place in gout, and every practitioner must have met with cases of erysipelas, which have required the extremes of opposite practice, in proportion as the affection has occurred to a strong and vigorous, or a broken and debilitated constitution; and as the head or the extremities may have been the subject of the attack. One plan of treatment cannot be made applicable under all the varying circumstances of *any* disease; and least of all in such a one as the gout, which appears under such a variety of forms as to have given rise to so many different appellations, as *atonic, retrocedent, misplaced, &c.* Almost every particular case of this disease is an exception from a general rule, and consequently will require its appropriate mode of treatment.

For your number for October, 1802, I sent you a paper on the subject of this disease, which had then occupied for several years, and still continues to occupy, a considerable portion of my attention. On that occasion I alluded to diminished temperature, which has ever made a considerable part of my practice in the first attacks of the gout, and to the younger part of my patients, where the constitution and activity of the vascular system have been vigorous. On the contrary, experience warrants me in declaring, that an opposite treatment is necessary in the cases of old debilitated habits, where the powers of life are languid, and the excitation of heat small. In such cases, instead of interfering with the regular formation of the paroxysm on the extremities by the indiscriminate abstraction of heat, every mean ought to be used to establish it there by such applications as not only tend to retain, but even in many cases to increase the quantum of heat in such parts. And this is done with the view of preventing parts of more consequence from being attacked, not by a translation of matter, but in consequence of that specific relaxed state of the parts, which constitutes the gouty diathesis, and which, in my opinion at least, renders them incapable of resisting the increased action of the vessels arising from nervous irritability. Though I do not deny that that many young persons, in whom the gouty inflammation (though widely different) approximates nearest to the common phlegmonous, may not only with impunity plunge their feet into cold water during a paroxysm of the disease, but even that they may frequently be relieved by doing so; yet this I strenuously contend for, that it is a practice fraught with mischief, if persisted in for a course of years. For, by accumulating the susceptibility of the parts, this practice in the first place not only renders the recurrence of the paroxysms more frequent, but subsequently lays the foundation of obstructions at an earlier period than would otherwise take place under a treatment that keeps the exhalents and absorbents of the parts in a state of greater freedom and activity.

As I have little leisure, I shall not enter farther at present into speculations on the subject; but after proposing to your ingenious correspondents the following questions towards a philosophical consideration of the subject, and pledging myself occasionally to take part in a *liberal* discussion of them, I shall proceed to state a few practical observations on the treatment of the disease.

First, I would ask the cause of animal heat in general? Secondly, The cause of the morbid excitation of it in a paroxysm of gout in particular? and Thirdly, Whether it be the cause or consequence of the paroxysm?

Though for the reasons above assigned, I am unfriendly to the application of cold water in a fit of the gout; on the other hand, I am no less so to the common mode of wrapping up the part in several folds of flannel. The true line of practice, as is usual in analogous cases, will be found somewhere between these two points; varying according to the peculiar state of the patient. A certain degree of heat is necessary to induce perspiration, which is generally allowed essential in promoting the most favourable solution of the paroxysm. We equally miss the true perspirable point, by covering the parts with many folds of flannel, and by immersing them in cold water. By the first we add greatly and unnecessarily to the present torments, and subsequent debility of the patient; by the latter we do no more than for the present almost extinguish the fire, by adding fuel hereafter to increase its heat.

As the gout appears to me a general and not a local disease, it follows that I cannot approve of local applications *only* for curing or preventing its paroxysms. Local applications ought to go hand in hand with general ones; and of the latter, gentle saline aperients and diaphoretics,

I have

I have uniformly found the best. I do not believe that hemlock, which I have seen used repeatedly, and the other narcotics, are entitled to the encomiums lately bestowed on them. In general, I find the best effects from doing something more than obviating costiveness with repeated doses of magnesia vitriolata and magnesia alba in peppermint water, carefully avoiding all irritation by carrying it so far as to purge, unless the excitement and heat of the system be very inordinate indeed. A grain or a grain and a half of ipecacuanha every six or eight hours, answers well as a diaphoretic. The temperature of the room and bed deserves much attention, as the excitement and generation of heat, except as before stated in persons in the last stage of life, require to be reduced rather than increased.

Sudden transitions from heat to cold, or cold to heat, ought equally to be avoided; and all changes of this nature should be brought about in the most gradual manner. The simple expansive power of heat is generally known. The mischief which must result from the sudden and alternate dilatations and contractions of the minute vessels of a part may be easily conceived.

With regard to external applications, none out of the many, which I have tried, have proved so generally effectual as steam, and occasionally confining the part for a while in a rarer atmosphere. I state this as a fact, the result of much experience; and apprehend, that this mode of treatment anticipates the tedious struggle which the parts affected must otherwise undergo, and disposes them to part with their heat, when excessive, more easily afterwards. This treatment not only has the happiest effects on the paroxysms while present, but renders subsequent ones more mild, and much protracts the intervals between them: and this in proportion to the prudent administration of it. I am much gratified in having possessed so much of the confidence of many judicious persons as to have enabled me to convince them of this truth; and I am little solicitous about others, who imagine that because they are not completely cured of the gout by two or three applications, they have given the plan a sufficient trial!!

I have several patients, who have by a steady use of the Air-pump vapour-bath every other or third day for a month at one time, and afterwards twice a week for the same period, and then by occasional applications, perhaps once or twice a month, have now been free from all appearance of gout for the last two years, and have not only enjoyed better general health, but have been competent to far more exertion than they had been for the two preceding years.

I beg leave to enclose you a letter of Dr. Stenhouse of Edinburgh, which I trust you will think deserving a place in your Journal: and I hope its having appeared in the Edinburgh Courant of the 9th of January last, will not operate as an objection.

---

"I have heretofore given some communications on the effects of ginger in the gout; and, although I have received much relief in the painful stage of that disorder, by the daily use of it for these three years past, yet the debility that followed was not less tedious; so that I continued my pursuit of something more efficacious, which I am hopeful I have at last found, and which I consider to be a duty to promulgate. Much have I thought for these eighteen years, and many an unintelligible page have I read upon this subject—but to come to the present question, since I am not writing a book. In the month of April last, a publication was put into my hand, which had escaped my notice, by a judicious acquaintance, to whom I am much obliged, entitled, "Facts and Observations respecting the Air-pump Vapour-Bath, in Gout, Rheumatism, Palsy, and other disorders, by Ralph Blegborough, M. D. Member of the Royal College of Surgeons, London;"—which apparatus, if it has all the effects ascribed to it, should be in every hospital and neighbourhood.—I was much pleased with the successful operation of this apparatus, because it confirmed an opinion I have long entertertained, of the immediate cause of a paroxysm of the gout, for of the gout I am only speaking, that I was determined to try the experiment on myself the first opportunity, though on a more simple scale; my opinion will be elucidated by the following remarks and experiments.

"The immediate cause of all acute pain I take to be either *irritation* or *obstruction;* the latter is surely the immediate cause of a gouty paroxysm. To trace causes to their elements is but an uncertain pursuit, and cannot be attempted here. That this obstruction takes place in the minute branches of the arteries, I hold to be true; nor do I see any phenomenon in a fit of the gout, but what may be accounted for by this hypothesis. It will be easy to see that for the present I deny the existence of gouty matter; nor do I consider the earthy concretions formed in the joints, after repeated severe attacks, to be a proof of this, since since the same phenomenon may be produced from the blood out of the body by a similar process. It is remarkable, that though much has been written on this subject, so little has been attempted, either to prevent the generating this disease, or mitigating the violence of its paroxysms. The reason of this I take to have been a supposition that there was something deleterious in the obstructed matter, and that it was unsafe either to prevent the fit, or tamper with the parts affected; of this prejudice I have had my share until within these three years. There is a prevalent opinion I know, with those unacquainted with the laws of the circulation of the blood, that there are applications, very improperly called repellants, which may drive back the gouty matter; but I tell my gouty readers, there is no operation can take place in the animal system in this sense; in fine, there can be no repellants, nor discutients, where there are no absorbents; but my readers must be cautious how they counteract the intensions of nature, or, if they must use the word, they must beware that by improper applications they do not *repel* the disposition of the system to produce a paroxysm, and thereby send it to some more vital part, which happened to myself the first symptom I had of this disease.

"I come now to describe my practice upon myself. I have already said, I took the hint from the Air-pump vapour-bath eight or nine months ago. The end of September last I was attacked in my right hand, but being in the country, I could not put my intentions in practice until I came home; by this time the fit had acquired its last stage both in pain and swelling. I then got a common tureen half full of boiling water; I laid my hand across, and covered it all over with some folds of flannel; but presently the steam was so hot, that I was obliged to reduce the heat of the water, so as to be able to bear the steam. In a few minutes the pain abated, and in about twenty-five minutes I was perfectly free from pain; and as the steam became so cold as to be no longer useful, I dried my hand and wrapt it up in flannel, and, had it not been for the swelling, I could have used it as well as if nothing had happened. About this time my right foot began to give me some symptoms of an attack; I allowed it to proceed for about 24 hours, until I was convinced it was to be a real fit. I then got a pail with two handles, and from the handles I suspended a towel to rest my heel upon; I then filled the pan with boiling water, so full as not to touch my heel, and covered it over with several folds of flannel for about

 half half an hour, as in the first experiment; I dried my foot, and wrapped it up in flannel; I was perfectly free from pain, and walked about the room as usual. I repeated this immersion five or six times this day and the following, since when I have had no complaint in my foot; but as I had only once immersed my hand in steam, in two days the pain returned, as if the obstruction had not been perfectly removed. I had recourse to the steam again, which I repeated two or three times. I have waited thus long to give a fair trial to its effects. I am still alive, and have been in good health ever since, though at the border of seventy.

"May I not fairly say, that here are two experiments, and what is more, at different stages of the paroxysms, which have been successful in removing the immediate cause, which I consider to be obstruction only, by the relaxing quality of the steam, or, what is the same thing, diminishing the pressure of the common atmosphere. Finally, I shall continue the ginger daily, and repeat the vapour bath when necessary; and if either stomach or bowels, or other viscera, should be attacked, I shall immerse my whole body in a hogshead of steam. To prevent the frequent return of the paroxysms, I live abstemiously, being certain that, in my case, the habit of body between repletion and inanition will conduce thereto; and such a state will be the most likely to prevent or mitigate diseases of any kind. If what has been said and done shall be thought erroneous, I shall kiss the rod of conviction." "A. STENHOUSE."

---

The above led to a correspondence between Dr. Stenhouse and myself; the following I copy from one of his letters received the other day. "I fancy I shall shortly have to appear again in print, to contradict a false report, namely, that I have retracted my opinion of hot vapour, and that I have had a severe relapse."

It is beyond every thing gratifying to me, to have my endeavours approved by a person so well qualified by education, great attention to the subject, and personal feeling, to judge of them as Dr. Stenhouse must be.

I trust, Gentlemen, these observations will have a tendency to prove that,

> "Est modus in rebus; sunt certi denique fines,

> Quos ultrà citràque nequit consistere rectum."

I am, &c.

RALPH BLEGBOROUGH.

*Margaret Street, Cavendish Square, June 15, 1804.*

